# Research Status and Trends in Virtual Reality Technology for Older Adults: Bibliometric and Visual Analysis

**DOI:** 10.2196/76609

**Published:** 2025-12-02

**Authors:** Jing Xu, Wenjin Zhang, WenLi Liu

**Affiliations:** 1 School of Nursing, Shandong Xiandai University, Jinan, China; 2 Shanxi Bethune Hospital, Shanxi Academy of Medical Sciences, Third Hospital of Shanxi Medical University, Tongji Shanxi Hospital, No. 99 Longcheng Street, Xiaodian District, Taiyuan, Shanxi, 030032, China, 86 13703519899; 3 Linfen Central Hospital, Linfen, China

**Keywords:** virtual reality, older adults, bibliometric analysis, CiteSpace, visual analysis

## Abstract

**Background:**

Virtual reality (VR) technology is increasingly applied in aging-related research. Although existing bibliometric studies have focused on specific applications, such as older adults’ acceptance of VR and its use in cognitive rehabilitation, no comprehensive mapping of the global research landscape on VR for older populations has been conducted. This study fills this gap by providing a holistic bibliometric and thematic analysis of VR applications in older adults, mapping research trends, intellectual structures, and emerging frontiers.

**Objective:**

This study aims to explore the current applications, potential benefits, and future directions of virtual reality technology for older adults, based on literature published between January 1, 2015, and April 30, 2025.

**Methods:**

This study used bibliometric methods to systematically examine the current status and developmental trends in VR research for older adults. We searched the Web of Science Core Collection for research articles and reviews published in English. A total of 1609 publications were included in the final analysis. Using CiteSpace and VOSviewer, we conducted coauthorship network analysis, keyword clustering, and burst detection to map research hot spots, academic collaboration patterns, and emerging trends in the field.

**Results:**

Our analysis of 1609 publications revealed a steady growth in the application of VR technology for older adults. The predominant research areas included meta-analysis, rehabilitation, dementia, and gait. The United States and China were the two most productive countries, with Tel Aviv University emerging as the leading institution. *Frontiers in Aging Neuroscience* and *Applied Sciences Basel* were the most prolific journals, each publishing 40 papers. The most cited article evaluated the effects of VR-based physical and cognitive training on executive function and dual-task gait performance in older adults with mild cognitive impairment. Emerging research themes include artificial intelligence, association, and depression.

**Conclusions:**

VR research for older adults is rapidly expanding and globally collaborative. Although applications span multiple geriatric domains, future efforts should prioritize mental health, disease integration, and artificial intelligence–enhanced VR technologies.

## Introduction

### Background

According to the World Health Organization, the global population aged 60 years and older reached 1.1 billion in 2023 and is projected to double to 2.1 billion by 2050 [1,2]. Declines in physical and mental health, loss of functional capacity, and social isolation hinder active aging among older adults. These factors not only threaten their quality of life but also pose significant challenges to global public health [3]. In this context, virtual reality (VR) has emerged as a promising interactive technology, demonstrating broad application potential in the field of older adults [4-8].

VR systems are defined as highly interactive 3D digital media platforms [9], with applications across diverse environments, including health care innovation [10]. They enhance user-environment interactions, offer real-time performance-based feedback, and improve accessibility and cost-efficiency. Empirical studies demonstrate their potential to prevent social isolation, encourage health and well-being, and enhance quality of life [11,12]. VR-related research is rapidly expanding, with advancements in VR-based rehabilitation and cognitive intervention yielding substantial clinical evidence [7,13-16].

However, systematic analyses of knowledge structures, trends, and evolutionary patterns in geriatric applications are still scarce. A critical gap exists in leveraging bibliometric and visualization techniques to map disciplinary collaboration networks and developmental trajectories within this domain.

In recent years, bibliometrics has become a key method for assessing academic literature using mathematical and statistical techniques [17,18], and it is recognized as a robust method for unveiling disciplinary development trajectories [19]. Such analyses provide data-driven insights that help researchers identify emerging research frontiers, trace disciplinary evolution, and refine strategic directions [20]. As the most widely used scientific citation database, Web of Science Core Collection (WoSCC) serves as a crucial resource for bibliometric data analysis.

This study used CiteSpace and VOSviewer for bibliometric and visual analysis. The analysis aimed to identify emerging trends and interdisciplinary connections, as well as to examine the impact of VR as a multifunctional intervention tool on research priorities and clinical translation. Specifically, we identified key research hot spots, influential authors, leading institutions, and evolving trends in this field.

### Objectives

This study aims to provide a more comprehensive understanding of VR applications in aging research by integrating multiple analytical dimensions, extending beyond the scope of prior bibliometric reviews. The findings are intended to guide researchers and practitioners in the development and implementation of VR technologies for older adults.

## Methods

### Data Sources and Search Strategy

We searched the WoSCC on May 7, 2025, and completed the search in one day to avoid bias from database updates. The search formula was “TS=(“virtual reality” OR “virtual medicine” OR “augmented reality” OR “mixed reality” OR “virtual simulation”) AND TS=(“older” OR “older adults” OR “elderly” OR “elder person” OR “older people” OR “oldest old” OR “elderly people” OR “elderly patient”). The search was restricted to articles and review articles published in English from January 1, 2015, to April 30, 2025.

We focused our literature screening on the application of VR technology to older adults, reviewing both abstracts and full-text articles. Duplicate and irrelevant topics were systematically removed from the dataset. The exclusion criteria also specified source types, including conference papers, reviews, data papers, edited materials, and retractions. After the initial search, all literature was screened and assessed separately by 3 researchers (JX, WZ, and WL) to ensure the relevance of the selected papers to the research topic. Any disagreements during the analysis process were resolved through internal discussions within the research team and consultations with experts to reach a consensus.

### Analysis Tools

In this study, Microsoft Excel (2013; Microsoft Corp) was used for organizing data and performing statistical analyses on the retrieved bibliometric data, including the generation of charts that depict the quantity and growth trends of publications. For the visualization and bibliometric analysis, CiteSpace (version 6.4.R2) and VOSviewer (version 1.6.20) were used to examine various aspects: author productivity and collaboration patterns, journal publication volumes, national and institutional cooperation networks, highly cited references, burst keywords, and keyword co-occurrence patterns. This comprehensive approach has facilitated an in-depth overview of the research progress and developmental trajectories concerning the application of VR technology among the older adult population.

### Ethical Considerations

The data were retrieved from WoSCC. This study involved no direct patient or public participation, as it was based solely on published literature. This study was conducted and reported in accordance with the BIBLIO (Guideline for Reporting Bibliometric Reviews of the Biomedical Literature) guidelines (Checklist 1). The recommendations of BIBLIO were followed to ensure full compliance with the applicable research design and reporting framework.

## Results

### Literature Screening Workflow

**Figure 1.**
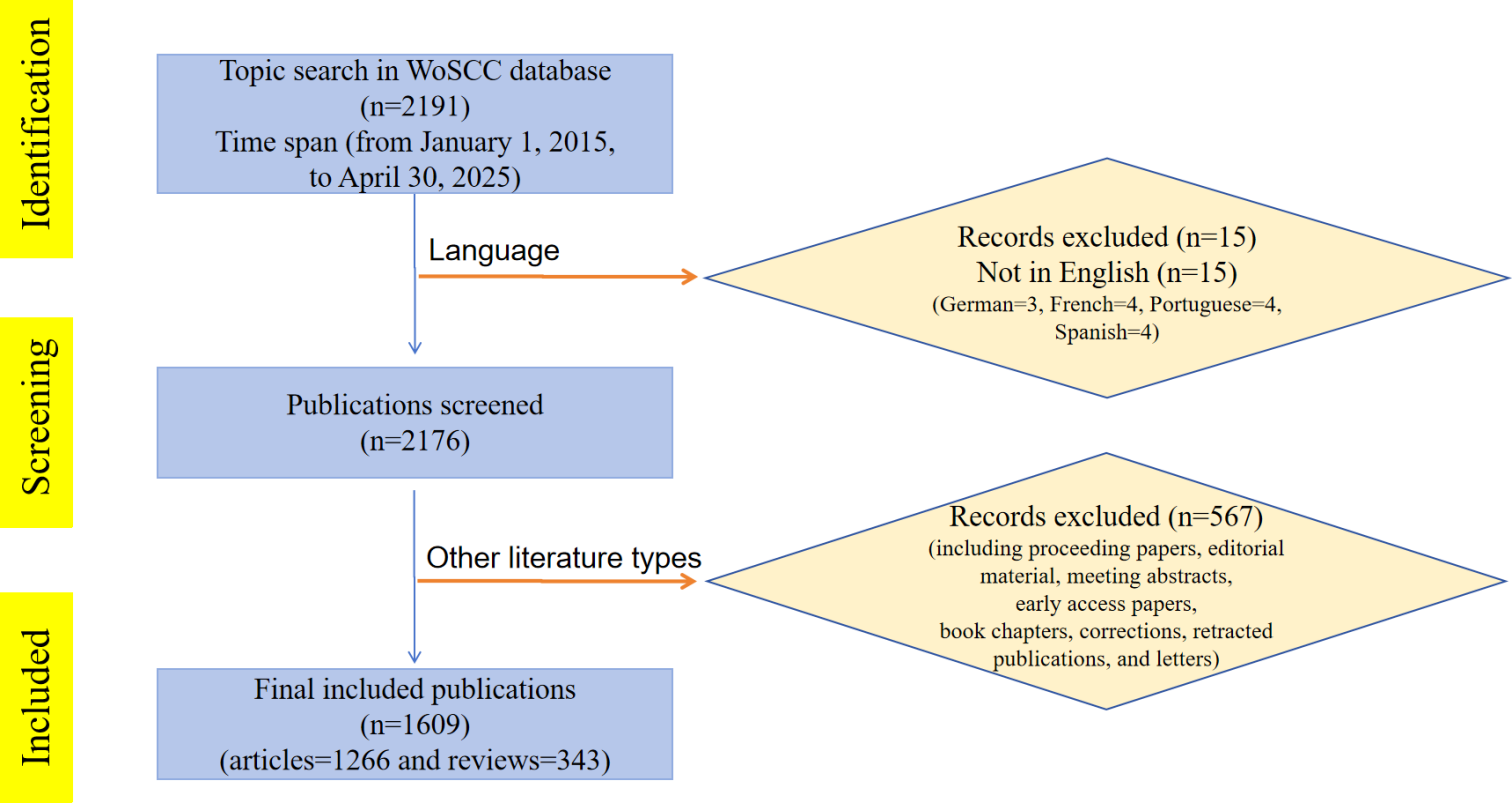
Literature screening workflow for the bibliometric analysis of virtual reality applications in older adults. WoSCC: Web of Science Core Collection.

Ultimately, 1609 articles met the inclusion criteria for this study. These articles were exported in their entirety, including full records and cited references, and saved as plain-text files in .txt format. The process of searching and filtering is illustrated in Figure 1.

### Number of Publications and Growth Trend

**Figure 2.**
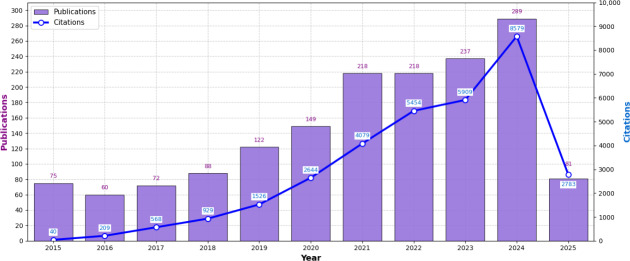
Trends in annual publications and citations on virtual reality applications for older adults. Global bibliometric analysis showed a peak of 289 publications in 2024; note that the 2025 data includes up to April 30.

The number of publications on VR applications for older adults has demonstrated consistent growth since 2022, reaching its peak at 289 articles in 2024 (Figure 2). This upward trajectory reflects a growing scholarly interest in VR technologies for aging populations worldwide.

### Principal Authors and Coauthorship Trends

**Figure 3.**
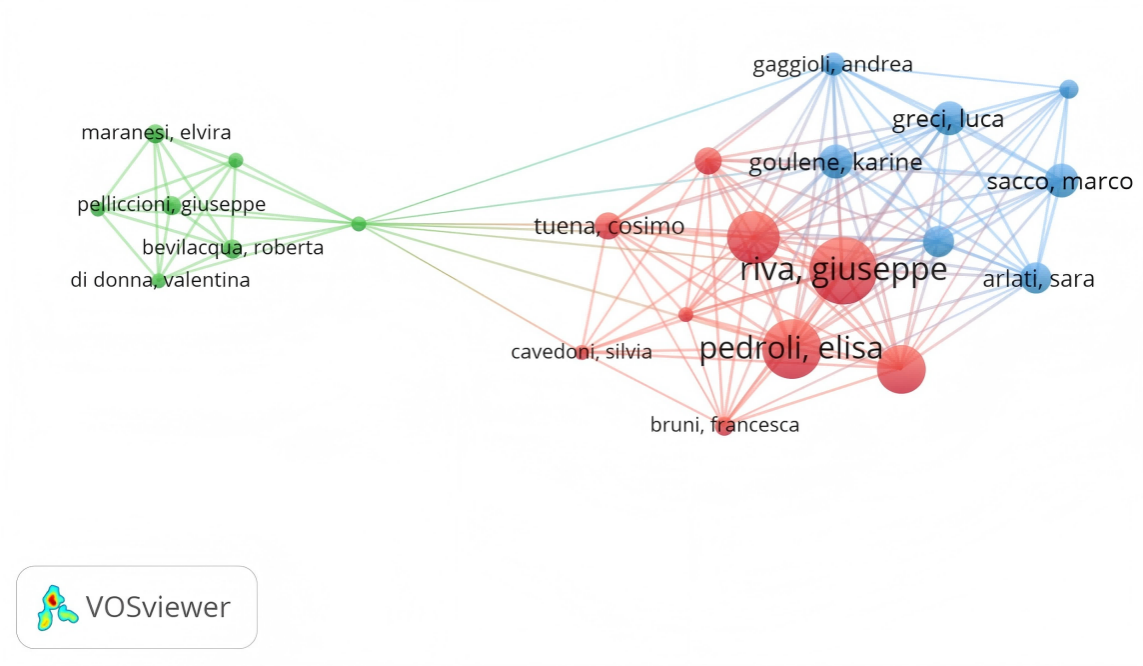
Global author collaboration network in virtual reality research for older adults.

Our research identifies that a total of 7291 authors contributed to the study of VR for older adults from January 1, 2015, to April 30, 2025. Furthermore, our observations suggest that there are particularly prolific authors who have established a collaborative network (Figure 3), indicating the development of several high-output research teams in this area.

### Publication Journal of VR Research for Older Adults

Our bibliometric analysis revealed that VR-related gerontological research is widely distributed across multiple academic journals. As shown in Table 1, the top 5 most productive journals collectively published 192 articles, accounting for 11.93% of the total publications in this field. *Frontiers in Aging Neuroscience* and *Applied Sciences Basel* led the group, with 40 publications each on VR applications for aging populations, followed by the *International Journal of Environmental Research and Public Health*, which had 39 articles.

**Table 1.** Top 5 journals ranked by publication count (as of April 30, 2025).

Number	Journal name	Country	Number of papers	Number of citations	Average citations per publication
1	*Frontiers in Aging Neuroscience*	Switzerland	40	1144	28.6
2	*Applied Sciences Basel*	Switzerland	40	332	8.3
3	*International Journal of Environmental Research and Public Health*	Switzerland	39	768	19.69
4	*Scientific Reports*	England	37	589	15.93
5	*JMIR Serious Games*	Canada	36	693	19.25

### Analysis of Countries and Institutes

**Figure 4.**
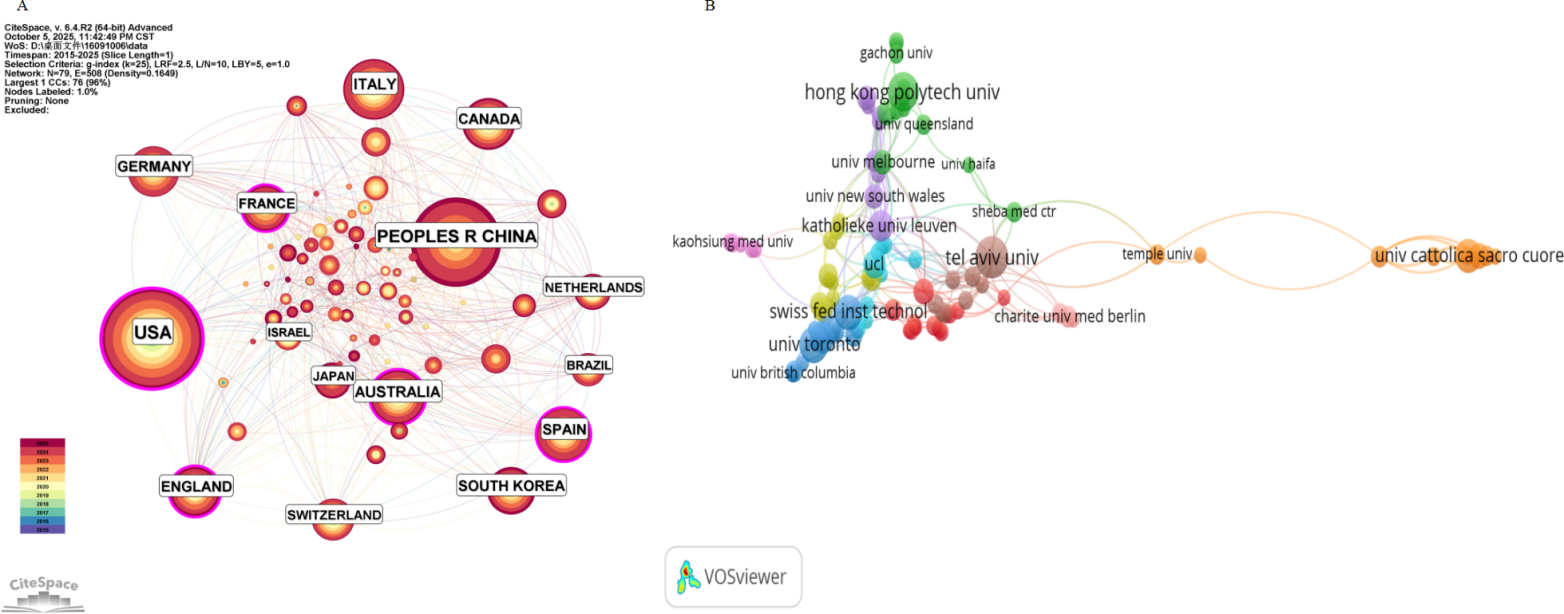
National and institutional contributions. (A) Distribution of publications from different countries. (B) Global institutional collaboration network in virtual reality research for older adults.

Our bibliometric analysis reveals contributions from 79 countries/regions and 2452 institutions, collectively producing 1609 publications. As illustrated in Figure 4A, the collaborative network highlights the United States as the leading contributor (n=338), followed by China (n=289) and Italy (n=134). The visualization reveals strong research collaborations between the United States and other leading nations, including Australia, Switzerland, and England.

Institutional analysis showed that the top 10 institutions account for 185 publications (11.5% of the total), with Tel Aviv University being the most productive (n=27), followed closely by Hong Kong Polytechnic University (n=25). Figure 4B demonstrates increasingly strong interinstitutional connections.

### Literature Cocitation and Cluster Analysis

**Figure 5.**
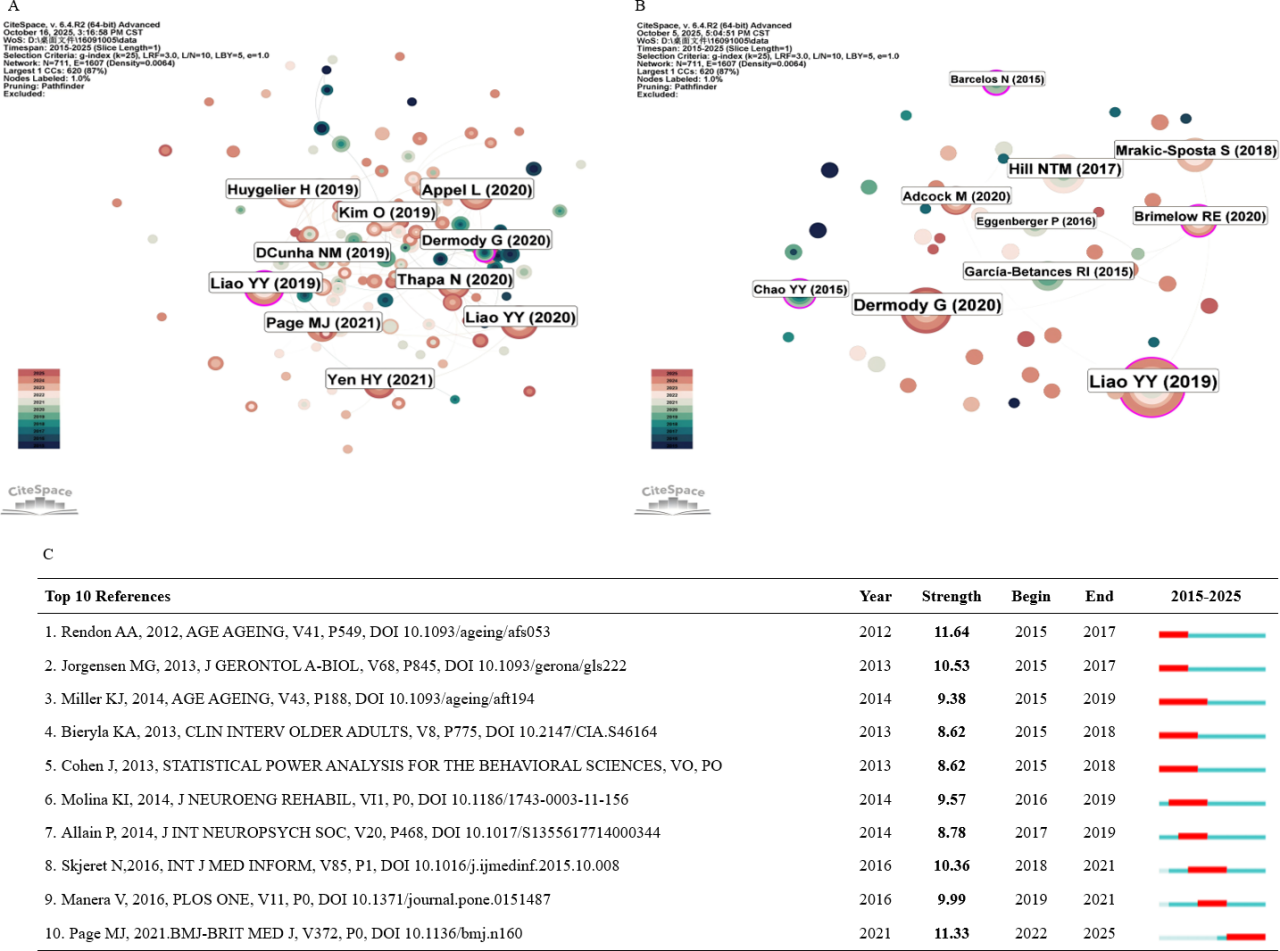
An analysis of the literature’s impact on the field. (A) Cocitation network visualization based on citation frequency. (B) Centrality map identifying the most influential papers. (C) Top 10 references with the strongest citation bursts.

A citation is a vital bibliometric indicator, with frequently cited studies greatly inﬂuencing their research areas [14]. Citation analysis serves as a critical bibliometric tool for assessing scholarly impact, where highly cited publications effectively identify research priorities within a field [21]. Table 2 lists the top 10 most influential articles ranked by total citation count and provides a visual analysis of their citation frequencies, as illustrated in Figure 5A.

**Table 2.** The top 10 most-cited articles on virtual reality–based interventions in older adults, ranked by citation frequency.

TC^a^	Title	Authors	Year	Journal	DOI
63	Effects of virtual reality-based physical and cognitive training on executive function and dual-task gait performance in older adults with mild cognitive impairment: a randomized control trial [22]	Liao et al	2019	*Frontiers in Aging Neuroscience*	10.3389/fnagi.2019.00162
63	Using virtual reality-based training to improve cognitive function, instrumental activities of daily living and neural efficiency in older adults with mild cognitive impairment [23]	Liao et al	2020	*European Journal of Physical and Rehabilitation Medicine*	10.23736/S1973-9087.19.05899-4
60	Older adults with cognitive and/or physical impairments can benefit from immersive virtual reality experiences: a feasibility study [24]	Appel et al	2020	*Frontiers in Medicine*	10.3389/fmed.2019.00329
57	The effect of a virtual reality-based intervention program on cognition in older adults with mild cognitive impairment: a randomized control trial [25]	Thapa et al	2020	*Journal of Clinical Medicine*	10.3390/jcm9051283
51	The effectiveness of virtual reality for people with mild cognitive impairment or dementia: a meta-analysis [26]	Kim et al	2019	*BMC Psychiatry*	10.1186/s12888-019-2180-x
47	Virtual reality exergames for improving older adults’ cognition and depression: a systematic review and meta-analysis of randomized control trials [27]	Yen and Chiu	2021	*Journal of the American Medical Directors Association*	10.1016/j.jamda.2021.03.009
47	PRISMA 2020 explanation and elaboration: updated guidance and exemplars for reporting systematic reviews [28]	Page et al	2021	*BMJ* (*British Medical Journal*)	10.1136/bmj.n160
46	Acceptance of immersive head-mounted virtual reality in older adults [29]	Huygelier et al	2019	*Scientific Reports*	10.1038/s41598-019-41200-6
45	A mini-review of virtual reality-based interventions to promote well-being for people living with dementia and mild cognitive impairment [30]	D’Cunha et al	2019	*Gerontology*	10.1159/000500040
43	The role of virtual reality in improving health outcomes for community-dwelling older adults: systematic review [31]	Dermody et al	2020	*Journal of Medical Internet Research*	10.2196/17331

a TC: total number of citations.

In network analysis, betweenness centrality reflects the importance of nodes within a network; higher values indicate greater influence [32]. Figure 5B presents the top 10 publications ranked by betweenness centrality. A visual analysis, with pink nodes highlighting those with high betweenness centrality, reveals key structural influencers in the citation network. The study by Barcelos et al [33], published in the *Journal of the International Neuropsychological Society*, exhibited the highest betweenness centrality (0.25), followed by Brimelow et al in *Cyberpsychology, Behavior, and Social Networking* [34] (0.12) and Liao et al in *Frontiers in Aging Neuroscience* [22] (0.11). These publications serve as central hubs for knowledge dissemination in VR research for older adults, helping to identify and anticipate key developments and emerging trends in the field.

Additionally, we analyzed citation burst strength and identified the top 10 articles with the strongest bursts (Figure 5C). The study by Rendon et al [35], published in *Age and Aging*, exhibited the highest burst strength (11.64). The article by Page et al [28], published in *BMJ*, also showed a strong citation burst (11.33), reflecting rapid academic attention.

### Keyword Visualization and Cluster Analysis

**Figure 6.**
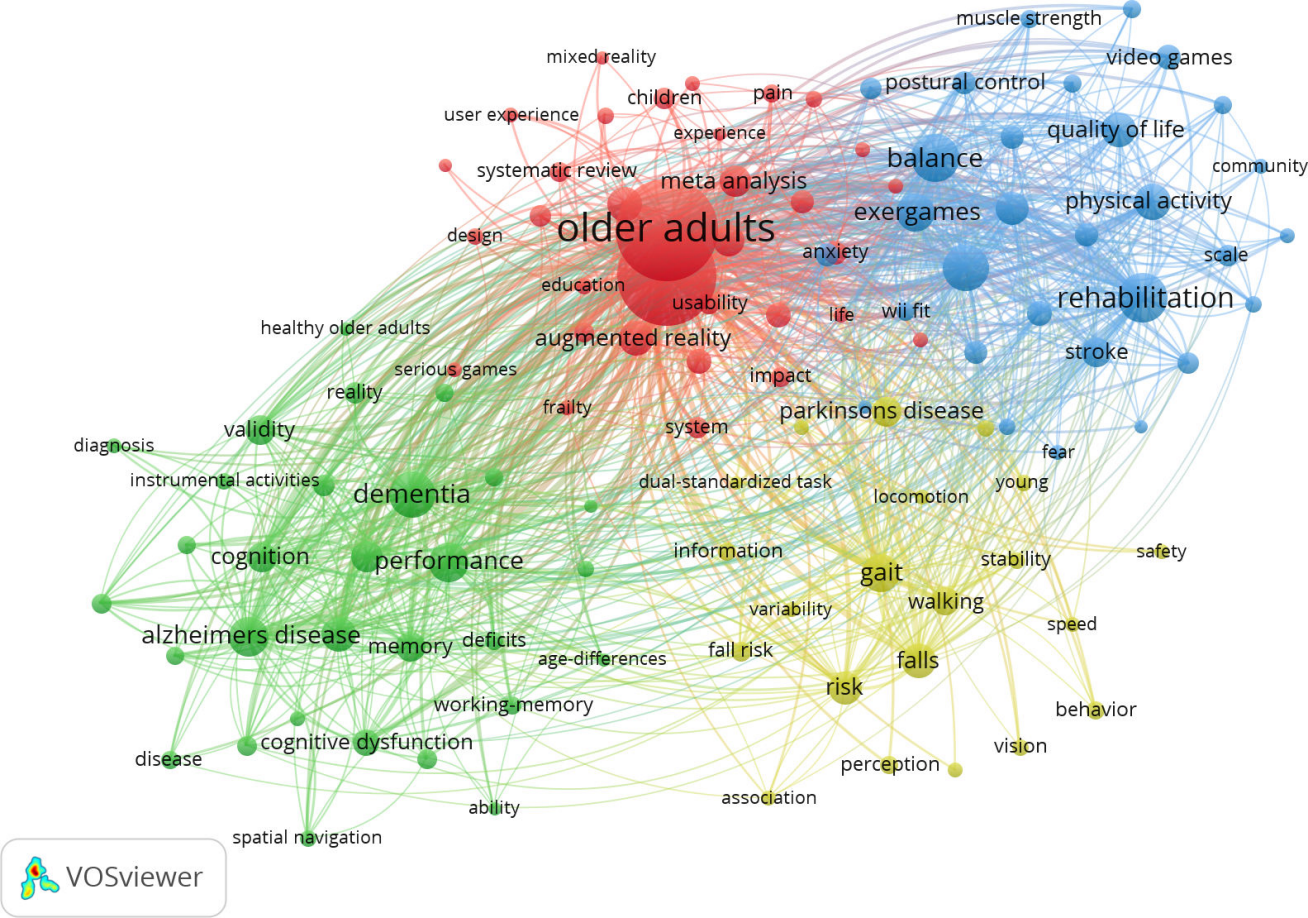
Keyword co-occurrence network analysis (2015‐2025) of 1609 articles on virtual reality for older adults.

Through a systematic keyword analysis of 1609 publications using VOSviewer and CiteSpace, 4 key research clusters were identified in the application of VR for older adults (Figure 6). Cluster 1 focuses on meta-analysis, emphasizing methodological synthesis and evidence-based approaches. Cluster 2 is characterized by terms such as rehabilitation, balance, and exergames, highlighting the role of VR in improving physical function and mobility. Cluster 3 includes keywords like dementia, Alzheimer disease, and cognition, indicating the potential of VR in the diagnosis and management of neurodegenerative conditions. Cluster 4, marked by terms such as gait, falls, and stability, addresses the utility of VR in fall prevention and gait rehabilitation. Together, these clusters outline the current research landscape of VR applications for older adults.

**Figure 7.**
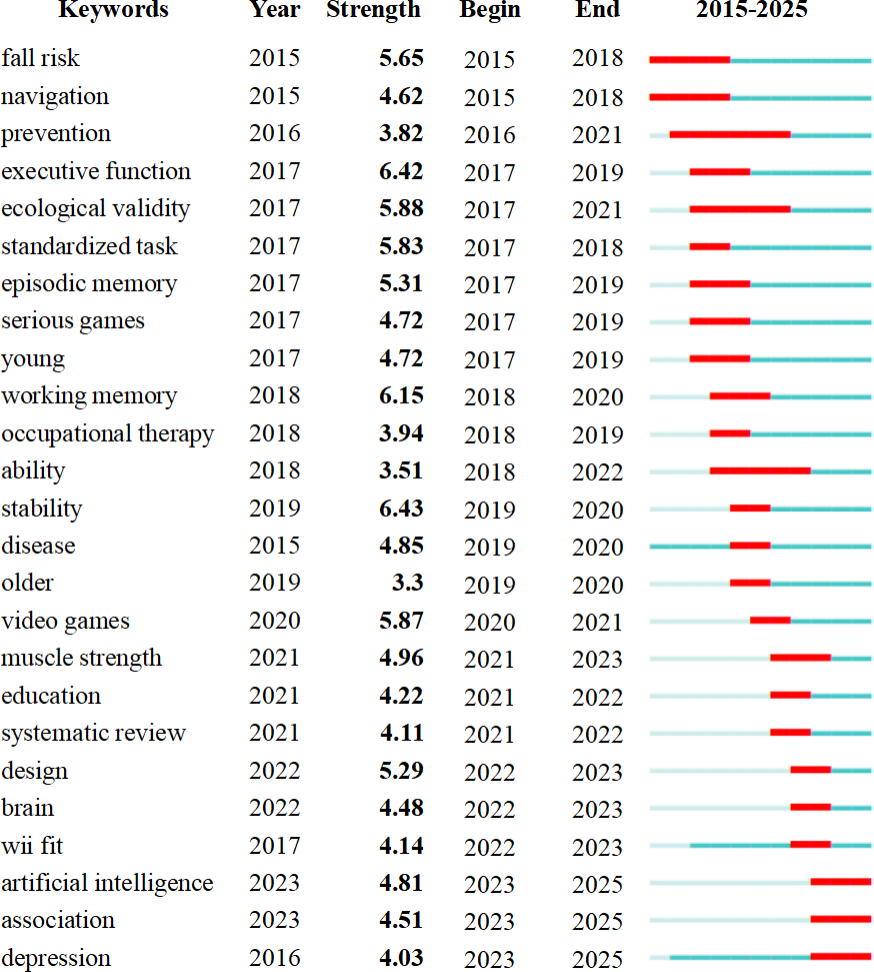
Keyword burst detection in virtual reality research for older adults: identification of emerging research fronts.

The identification of keyword bursts, which refer to sudden increases in citation frequency over short periods, serves as a critical indicator of emerging research frontiers [36]. Figure 7 presents the 25 most prominent keyword bursts from 2015 to 2025, with thick red bars indicating periods of heightened activity. The term “stability” exhibited the strongest burst intensity (6.43), followed closely by “executive function” (6.42). Notably, “prevention” showed the longest sustained burst duration (6 y). Recently emerged keywords include “artificial intelligence,” “association,” and “depression,” collectively reflecting current research trajectories that may shape future investigative priorities in this field.

## Discussion

### Principal Findings

The accelerating global demographic shift toward population aging has elevated geriatric health to a pivotal concern at the intersection of public health and technological innovation. Our bibliometric analysis of 1609 scholarly publications revealed that research on VR applications for older adults has evolved from isolated intervention trials into a well-defined, globally collaborative, and rapidly expanding field characterized by sustained growth and increasing interdisciplinary integration. Persistent geographical disparities in research advancement call for transnational alliances and structured knowledge exchange, strengthened collaboration between institutions in developing and advanced countries, and equitable co-development of VR technologies for aging.

We conducted a cocitation analysis to map the knowledge structure and thematic evolution of the field. The results revealed a dual emphasis on cognitive and emotional health in VR applications for older adults. Liao et al [22] demonstrated in a randomized controlled trial that VR improves executive function and dual-task gait in individuals with mild cognitive impairment, highlighting its potential for cognitive rehabilitation. Brimelow et al [34] used a mixed methods design in nursing homes and found that immersive VR significantly reduces apathy, underscoring its value as a psychosocial intervention.

The keyword co-occurrence network revealed 4 distinct clusters, reflecting the evolving research landscape of VR applications for older adults. Notably, the red cluster captured a pivotal shift in the field—from initial exploration of clinical feasibility to a growing emphasis on methodological rigor and evidence synthesis.

This transition was further corroborated by citation burst analysis of references, which showed that early research was dominated by studies on VR’s efficacy in balance improvement and fall prevention [35], reflecting a formative phase focused on proof-of-concept and immediate outcomes. In contrast, recent citation surges highlighted methodological frameworks and reporting standards—particularly PRISMA (Preferred Reporting Items for Systematic Reviews and Meta-Analyses) [28]—indicating a maturation toward transparent, reproducible, and evidence-informed research practices.

Systematic reviews provide comprehensive and transparent evaluations of existing literature, supporting clinical decision-making and guiding future research directions. For instance, a recent systematic review of commercially available VR applications found that VR interventions hold potential for improving health outcomes in older adults [31]. However, the studies included in such reviews generally did not reach high certainty or quality according to the GRADE (Grading of Recommendations Assessment, Development and Evaluation) framework, and authors emphasized the need for more rigorous scientific methodologies to robustly evaluate and validate VR technologies.

The blue cluster centered on rehabilitation, balance, and exergames, reflecting VR’s role in addressing physical and cognitive decline in older adults. Exergames—VR systems combining exercise with gamified feedback—have been integrated into cognitive training with promising engagement [37,38]. Yet, a systematic review found limited high-quality evidence of sustained benefits in long-term care settings [39], underscoring the need for larger, rigorous trials that consider cost-effectiveness and user-centered design.

The green cluster focused on cognitive decline and dementia, highlighting an emerging trend in the application of VR for neurocognitive rehabilitation. VR technology offers immersive, simulated environments that can replicate real-life scenarios, providing patients with safe, controlled, and repeatable opportunities for functional training. A recent meta-analysis further confirmed the dual benefits of VR: not only does it enhance cognitive processing and sensorimotor coordination, but it also positively promotes mental health by increasing user engagement and autonomy [40].

The yellow cluster underscored VR’s role in enhancing mobility and preventing falls in older adults. VR-based interventions simulate dynamic environments, enabling safe and controlled training of adaptive motor responses. Evidence shows these immersive experiences improve gait and dynamic balance in older adults with Parkinson disease through engagement in rich, complex, and personalized virtual scenarios [41].

### Research Hot Spots and Frontiers

Burst keyword analysis in this study revealed that the first prominent burst was associated with AI, which demonstrated a strong growth trend from 2023 to 2025. This indicates that artificial intelligence (AI) is emerging as an increasingly central driver in aging-related research. AI encompasses core technologies such as machine learning, natural language processing, and computer vision, all of which show significant potential in addressing unmet needs among older adult populations.

For instance, innovative systems integrating AI and VR have been developed to create immersive social virtual environments that simulate interpersonal interaction scenarios, aiming to improve emotional well-being and cognitive function in older adults [42]. Additionally, AI-powered transformations of hospital waiting rooms—enabled by the integration of Internet of Things and AI technologies—are being explored to enhance health care efficiency, optimize patient experience, and support early disease detection [43]. Recently, a study introduced an adaptive AI-driven content generation system based on user feedback, capable of dynamically adjusting the difficulty and thematic content of cognitive training tasks. This system delivers personalized and engaging experiences for older users, effectively slowing cognitive decline and significantly improving quality of life, emotional stability, and subjective well-being [44].

In summary, future research should further advance the embedded application of AI within VR environments, with particular emphasis on two critical directions [3]: (1) ensuring safety and privacy protection mechanisms for AI systems deployed in home settings and (2) bridging the “digital divide” by addressing older adults’ technological barriers and trust concerns in using AI-driven tools, thereby promoting equitable access to digital health innovations.

The keyword “association” showed a significant increase during 2023-2025, reflecting growing scholarly attention to the relationships between VR interventions and health outcomes in older adults. This trend indicates a shift in research focus—from primarily validating the efficacy of interventions toward exploring underlying influencing factors and mechanistic pathways. This transition is empirically supported by Oliveira et al [45], who developed the ECO-VR multitask assessment system. Their findings revealed that task performance was significantly associated with age, education level, Mini-Mental State Examination scores, and verbal fluency, with age emerging as the strongest predictor. This underscores the critical role of demographic variables in shaping VR-based outcomes. The study further confirms that VR can serve as an ecologically valid tool for assessing multidimensional cognitive associations in aging populations.

This growing emphasis on association aligns with the broader trend in digital health toward personalization and precision medicine. As AI and machine learning are increasingly integrated into VR systems, it becomes feasible to capture user behavioral data in real time and identify dynamic associations with cognitive decline or fall risk. Predictive models derived from large-scale data analysis may uncover causal pathways between clinical indicators, enabling early risk detection and timely intervention.

The third research hot spot is depression, characterized by persistent sadness and loss of interest in daily activities, necessitating early detection for effective treatment and intervention [46]. It significantly impairs quality of life, physical health, and cognitive function, while elevating risks of social isolation, suicidal ideation, and health care utilization [47]. This burden is particularly pronounced among long-term care residents, where environmental constraints, loneliness, and limited social engagement act as key contributing factors [48]. In this context, VR technology has emerged as a promising nonpharmacological intervention [27,49,50].

### Challenges to Clinical Translation

Despite growing academic interest in VR for older adults, significant translational barriers persist in implementing VR within community and home-based care settings. These barriers operate across 3 interconnected domains. First, the absence of a standardized scientific framework for developing and evaluating VR-based interventions impedes evidence-based implementation. Second, high equipment costs and low digital literacy limit VR adoption among older adults [51], while implementation costs often exceed marginal health benefits [52]. Third, commercial VR platforms often feature complex interfaces and immersive environments, which may pose potential safety risks.

First, establishing a standardized scientific framework for VR-based therapy development and evaluation [53] is essential. Concurrently, deploying voice-guided VR interfaces on low-cost or refurbished devices could mitigate financial and usability barriers. Technological innovation must be integrated with real-world economic constraints, ensuring designs align with older adults’ needs and preferences—empowering them to independently engage with technology [54]. Alternatively, family-mediated device guidance may enhance user experience and reduce adoption barriers.

### Limitations and Future Directions

Our study has several limitations that should be acknowledged. First, literature retrieval based on topic fields relies on partial keyword matching, which may retrieve records with weak relevance to the research topic, thereby introducing noise and compromising retrieval accuracy. Second, the restriction to English-language publications and specific document types may have introduced selection bias and limited the representativeness of the findings. Third, the reliance on a single database and a fixed time window constrains the comprehensiveness and generalizability of the results. Finally, automatic term extraction in CiteSpace and VOSviewer may have resulted in synonym redundancy or conceptual overlap, potentially affecting the accuracy of topic clustering. Future studies should refine search terms, use multidatabase sourcing, and validate keywords to enhance reliability.

### Conclusions

Addressing health challenges associated with population aging has become a critical component of global health strategies. Although VR technologies demonstrate considerable promise for geriatric applications, rigorous empirical investigations remain essential to establish the efficacy and feasibility of various intervention approaches. Future studies should prioritize the development of more targeted VR applications while systematically evaluating their adaptability across diverse older adult populations. Furthermore, enhanced international collaboration among scholars and institutions is warranted to accelerate the development and implementation of VR-AI integrated technologies, collectively addressing global challenges posed by population aging.

## Supplementary material

10.2196/76609 Checklist 1 The BIBLIO checklist for reporting bibliometric reviews of the biomedical literature.

## Abbreviations

AI: artificial intelligence
BIBLIO: Guideline for Reporting Bibliometric Reviews of the Biomedical Literature
GRADE: Grading of Recommendations Assessment, Development and Evaluation
PRISMA: Preferred Reporting Items for Systematic Reviews and Meta-Analyses
VR: virtual reality
WoSCC: Web of Science Core Collection
